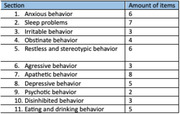# Correlations between monoaminergic biomarkers, classical biomarkers and the BPSD‐DSII behavioral scale in Down individuals with and without Alzheimer's dementia

**DOI:** 10.1002/alz.092217

**Published:** 2025-01-09

**Authors:** Charlotte Jacob, Hanane Kachar, Annelies Heylen, Marleen Tollenaere, Inge M.W. Verberk, Charlotte Teunissen, Peter Paul De Deyn, Debby Van Dam

**Affiliations:** ^1^ University of Antwerpen, Antwerpen, Antwerpen Belgium; ^2^ Neurochemistry Laboratory, Department of Clinical Chemistry, Vrije Universiteit Amsterdam, Amsterdam UMC location VUmc, Amsterdam Netherlands; ^3^ Neurochemistry Laboratory, Amsterdam UMC, Amsterdam Netherlands; ^4^ University of Groningen, University Medical Centre Groningen, Groningen Netherlands

## Abstract

**Background:**

Down syndrome (DS, trisomy 21) is the most frequent genetic cause of intellectual disability (ID), prevalent in approximately 1 in 900 live births (Loane et al., 2013). People with DS are at high risk to develop Alzheimer’s disease dementia (AD) (Lott & Head, 2001). Onset of clinical symptoms varies substantially in time. Consequently, predicting and monitoring decline and onset of dementia is a diagnostic challenge, while it is of essence in daily care and support. Behavioral and Psychological Symptoms of Dementia are an important and easily accessible tool for the prediction of dementia onset in the DS population. The BPSD‐DSII scale was developed to identify behavioral changes between the last six months and pre‐existing life‐long characteristic behavior in an (AD‐)DS population. Changes are assessed with the use of different behavior‐specific questions (items) which are categorized in different clusters (sections) (Dekker et al., 2018). We used the BPSD‐DSII to assess these symptoms and predict a potential diagnosis of dementia. BPSD‐DSII results are correlated with the serum levels of monoaminergic (epinephrine, norepinephrine, dopamine, serotonin and their metabolic products) and classical biomarkers, such as Ab40, Ab42, total tau, phosphorylated tau and neurofilament light chain.

**Method:**

We used reversed‐phase ultra‐high performance liquid chromatography with electrochemical detection to determine the levels of epinephrine, norepinephrine, dopamine, serotonin and their metabolic products in serum. The Simoa platform was applied to detect the levels of Ab40, Ab42, total tau, phosphorylated tau and neurofilament light chain. Finally, we worked together with the caregivers of the (AD‐)DS individuals for the collection of BPSD‐DSII data.

**Result:**

Preliminary data suggests correlations between different sections of the BPSD‐DSII questionnaire and serotonin levels, as well as correlations between different sections of the BPSD‐DSII questionnaire and classical biomarker levels. In addition, complementary data regarding the correlation between the different items of the BPSD‐DSII questionnaire and the different serum‐based biomarkers will be presented at the conference.

**Conclusion:**

The results per section of the BPSD‐DSII correlate with the levels of monoamines and classical biomarkers in (AD‐)DS individuals. And could therefore be used as potential biomarkers for the development of AD within the population of DS patients.